# Development of Low-Weight and High-Strength AA6005A Extrudates Intended for Modern Architecture and Design of Innovative Die for Extrusion Process

**DOI:** 10.3390/ma17102437

**Published:** 2024-05-18

**Authors:** Jacek Madura, Sandra Puchlerska, Maciej Balcerzak, Piotr Noga, Marek Bogusz, Józef Zasadziński, Dariusz Leśniak, Krzysztof Żaba, Henryk Jurczak

**Affiliations:** 1 Department of Metal Working and Physical Metallurgy of Non-Ferrous Metals, Faculty of Non-Ferrous Metals, AGH University of Krakow, al. Adama Mickiewicza 30, 30-059 Krakow, Poland; spuchler@agh.edu.pl (S.P.); balcerzak@agh.edu.pl (M.B.); bogusz@agh.edu.pl (M.B.); zas@agh.edu.pl (J.Z.); dlesniak@agh.edu.pl (D.L.); krzyzaba@agh.edu.pl (K.Ż.); 2 Department of Materials Science and Engineering of Non-Ferrous Metals, Faculty of Non-Ferrous Metals, AGH University of Krakow, al. Adama Mickiewicza 30, 30-059 Cracow, Poland; pionoga@agh.edu.pl; 3 Albatros Aluminum Corporation, 78-600 Wałcz, Poland; h.jurczak@albatros-aluminium.com

**Keywords:** thin-walled aluminum extrudates, aluminum extrusion, porthole dies

## Abstract

In the realm of modern architecture, the demand for materials that combine strength, durability, and aesthetic flexibility is ever-growing. Addressing this need, this paper presents a study on the innovative use of aluminum extrudates in construction. Focusing on the AA6005 alloy, which is known for its excellent balance of strength, corrosion resistance, and weldability, this research delves into the development of an extrusion process that yields thin-walled, lightweight, yet high-strength structural components. Using FEM simulations, a new extrudate of the AA6005A was developed. It is compatible with standard façade systems, with high-strength properties and a weight reduced by 20% compared to that of conventional extrudates made of the AA6063 alloy. Using CAD engineering and FEM simulations of aluminum extrusion process, an innovative die was designed for the extrusion process, ensuring uniform flow of metal from the bearing and minimizing the elastic deflection of the die. This resulted in an increase in the extrusion velocity of thin-walled extrudate from AA6005A by 24% compared to conventional profiles extruded from AA6063. As part of the research, a trial test was carried out in production conditions and the quality of the extrudates was tested by 3D optical scanning, mechanical and structural properties tests, and microstructure observation.

## 1. Introduction

In the ever-evolving field of architecture and engineering, professionals are constantly looking for materials that not only meet stringent construction requirements, but also offer the versatility needed for creative expression. This task presents a myriad of challenges that must be overcome to achieve the delicate balance between functionality and aesthetics in today’s buildings. These challenges involve the need to use high-strength materials to withstand structural loads and environmental stresses over time without deformation or failure. Lighter materials are preferred to reduce loads on foundations and structures, especially in high-rise buildings, while maintaining strength. At the same time, there is an increasing focus on sustainability, which requires recyclable, sustainably sourced materials with a low carbon footprint. Cost-effectiveness is also key here and covers not only the initial expenses but also long-term maintenance and durability. Materials must also meet building regulations and standards for safety, fire resistance, and energy efficiency. 

Reducing the unit weight of extrudates by thinning the walls of hollow sections may result in a significant reduction in the strength and stiffness of the element and the entire structure. Therefore, high-component 6000 series alloys such as AA 6005A or AA6082 are subjected to increased amounts of extrusion to improve the mechanical properties of extrudates after the heat-treatment process (T6)—tensile strength of 260–300 MPa with an elongation of 6–9%. AA6005A is characterized by the best price-to-alloy content ratio (better than that of AA6082), and therefore the purchase cost is slightly higher than the standard AA6063. However, assuming the profile extrusion speed is 10 m/s with reduced unit weight, the economic balance of the change is fully justified [1].

The AA6005A alloy was selected for consideration due to the higher content of alloy additions such as Si and Mg compared to those found in the commonly used AA6063. This creates the possibility of achieving much higher strength parameters of the extrudate through heat treatment of the extrudate that is cooled on the press lay-out and artificially aged after the extrusion process. This allows for compensation for the negative impact of wall thickness reduction on the stiffness and strength of the extrudate through higher alloy properties. On the other hand, the use of a high-component alloy increases the flow stress of the metal in the extrusion process, which makes it much more difficult to uniformly fill the thin-walled and wide bearing during the process, which influences the geometric accuracy of the extrudate. These unfavorable process conditions require the application of significant pressure on the billet surface, generating high values of force parameters and, at the same time, limiting the extrusion speed. Extremely unfavorable conditions result in the high energy consumption and low efficiency of the process, as well as extremely difficult operating conditions of porthole dies. This work, which takes into account the current limitations of the process of extruding thin-walled shapes from the AA6063 alloy, aims to eliminate the defined inconveniences by using a die of a special design. The new die design should ensure a reduction in frictional resistance in the process by minimizing factors affecting the resistance to plastic flow and metal pressure on the tool surface and ports, while increasing the maximum possible extrusion velocity of the high-component AA6005A. Amid these challenges, AA6005A aluminum alloy emerges as a promising solution, providing an optimal balance between the economics of the extrusion process and mechanical properties, structural integrity, and design versatility [2].

This composition offers a blend of features that are beneficial in architectural applications. It exhibits a good strength-to-weight ratio comparable to that of mild steel, which is essential in structural applications that require both lightness and durability. The alloy’s excellent corrosion resistance makes it suitable for use in a wide range of environments, reducing the need for maintenance, especially for anodized elements. The alloy can be easily welded and formed into complex shapes, allowing for architectural innovation without compromising structural integrity. In addition, AA6005 alloy tolerates anodizing and other finishes well, providing architects with a wide range of aesthetic options to achieve the desired look and texture. Aluminum is fully recyclable, and the use of AA6005 alloy can contribute to sustainable building practices by minimizing waste and reducing the environmental impact.

Given its versatile properties, AA6005 alloy offers an effective solution to the multi-faceted challenges facing architects and engineers. Its uses range from structural elements such as beams and columns to aesthetic elements such as façades, sunshade curtain walls, and other architectural details. Extrudates of AA6005A not only meet the critical structural requirements of modern buildings, but also provide architects with greater creative freedom, enabling innovative designs that are sustainable, cost-effective, and aesthetically appealing [3].

Modern aluminum façade lamella systems installed on large-surface glass buildings walls create a functional and aesthetic façade. The main purpose of using this type of system, apart from architectural considerations, is to ensure the building has a favourable energy balance by controlling the insolation of the interior and protection against unfavourable weather conditions [4]. The systems structure consists of wide thin-walled aluminum extrudates. The complex cross section of the profiles includes technological elements and walls, ensuring rigidity [5]. This extrudates type is characterized by an unfavourable width-to-wall-thickness ratio, which causes inconvenience during the extrusion process. Furthermore, the overall dimensions of the final elements, which are up to 6 m long, make it difficult to ensure uniform quality properties along the extrudates [6]. The demand for lightweight and durable structural elements is constantly growing, and the product must meet increasing quality and strength requirements [7]. Therefore, an extremely important stage of planning the extrusion process of this type of elements is the optimization of the die design, as well as appropriately designed product properties. Optimizing the extrusion process can lead to aluminum profiles with uniform flow velocity and temperature, factors that are crucial for achieving high-quality end products with minimal defects [8]. By employing concurrent design processes and optimizing the interdependencies and feedback among tasks, aluminum profile extrusion product development can be made faster, more cost-effective, and of higher quality, addressing the intricate demands of modern architecture [9]. Simulation and optimization of the extrusion process can improve process parameters, billet preparation, and the die design, leading to efficient material flow, reduced waste, and lower production costs. This contributes to making the construction of aluminum wall façades more sustainable and economically viable [10]. Through simulation-based design processes, complex extrusion problems can be tackled, allowing for the creation of extruded aluminum products with enhanced mechanical properties and optimized shapes. This opens up new possibilities for innovative design in building façades [11].

The optimization of the extrusion process and the shape of aluminum wall façades is, therefore, a multifaceted approach that enhances product quality, design efficiency, process efficiency, and sustainability. It supports the construction industry’s move towards more innovative, cost-effective, and environmentally friendly solutions. Therefore, a research plan was created that allowed for comprehensive optimization of the process of extruding aluminum façade elements.

In the first stage of the research, comprehensive tests of the static–structural load on conventional extrudates in the façade system were carried out. Based on the obtained results and the requirements specified by the European standard, a new, improved extrudate geometry was developed with a reduced unit weight of 1 m of the product. FEM simulations confirmed that the profile with the new geometry made of EN AW-6005A alloy meets all requirements.

The complexity of the shape, the use of increasingly small wall thicknesses, high dimensional accuracy resulting from narrowed tolerances, and the high quality of the surface necessitate the use of advanced solutions in the field of manufacturing technology [12]. This is especially true nowadays, in the era of intensive industrial development, in which it is important to maximize the efficiency of the process, which translates into an increase in the competitiveness of the company. This makes it necessary to improve the current technology and to search for new solutions to meet market requirements. In the case of thin-walled hole extrudates—apart from technological parameters and billet properties—the design of the tool is crucial [13,14,15].

As part of the research, a prototype of new thin-walled extrudates of AA6005A alloy with reduced weight and increased strength properties was developed. The new geometry of the cross-section was developed on the basis of FEM computer simulations of static deformation and strength of a single extrudate and in the façade systems. The cross-sectional area was reduced by 16%, which lowered the weight of a single 6 m long element by 3.8 kg while maintaining the required stiffness and strength of the structure. This enabled the installation of systems that are longer than those that can currently be installed. 

As part of the research, a comprehensive technology for extruding a new profile with reduced mass from the EN AW-6005A alloy was developed. With the use of FEM numerical simulations, a new die set design and a set of process parameters were developed, ensuring the extrusion velocity reached the maximum level of 12 m/min, which is highly efficient for this type of extrudate with an unfavourable ratio of width to wall thickness. 

In the last stage, a trial test and complete validation of extruded products in variable velocity–temperature conditions were carried out. The results of an industrial trial test confirmed the high accuracy of the results of numerical simulations with the use of the developed material models. The developed technology was implemented in production conditions. A complete validation of the quality of the products was carried out on the basis of an optical geometry measurement system and the study of the microstructure of extrudates cross-sections.

The paper presents comprehensive research results on the development of a new product with significantly improved operational and environmentally friendly parameters as an alternative to standard extrudates used as an element of facade systems in construction. As a result, an extrudate compatible with standard façade elements was designed with extremely thin walls and an unfavourable ratio of width to wall thickness, which is a huge challenge from the point of view of the efficiency and economics of the extrusion process. Interestingly, this work also presents the results of developing an innovative die and extrusion technology, which was fully implemented and tested in production conditions. The high quality of extruded products using the developed technology was also confirmed in validation tests using advanced research methods, including geometric accuracy in optical scanning testing. The production process using an innovative die combined with appropriate cooling of extrudates on the lay out of the press and a dedicated heat-treatment process achieved significantly higher strength parameters of new products made of the AA6005A alloy by almost 18% compared to standard products made of the AA6063 alloy, which was confirmed by strength tests. The new technology ensures high efficiency of the extrusion process compared to that of a conventional die, which is unique in the current state of knowledge. While there are papers that have determined the impact of die design on the efficiency of extruding thin-walled extrudates from AA6005A, this mainly concerns profiles with relatively small dimensions where the ratio of the hollow extrudate width to the wall thickness is not problematic in terms of die design. The work presents a unique die design that ensures high production efficiency of 10–12 m/min, which is attractive even for smaller-sized profiles [7].

## 2. Materials and Methods

### 2.1. Development of Extrudate Cross-Section Geometry

The most commonly used wide extrudates for façade systems are extruded from AA6060 or AA6063, which are the subjects of ours tests. Standard extrudates are installed in the system in several variants. The method of installation determines the range of system functions—both rigid and movable systems are offered. Therefore, comprehensive static and strength simulations of the profile load in various variants of the support were carried out. The study was performed using Ansys Static-Structural software (v.R19.0, Ansys Inc., Canonsburg, PA, USA).

Ansys is a finite element analysis (FEA) software that is used to perform structural analysis using advanced solver options, including linear dynamics, nonlinearities, thermal analysis, materials, composites, and hydrodynamics. Finite Element Analysis is a numerical technique used to solve complex engineering problems related to structural mechanics, heat transfer, and fluid dynamics. It divides a complex structure into smaller, finite-sized elements that are easier to analyse.

**Figure 1:**
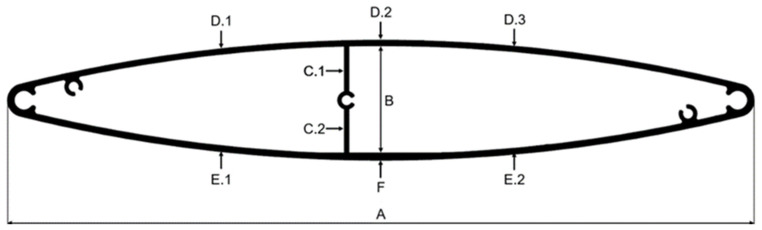
Cross-section geometry of standard extrudate intended for façade system.

The cross-section of standard profile is shown in Figure 1. The overall dimensions are 300 mm width (A) and 48 mm height (B), and all thicknesses range from 2.0 to 2.2 mm (C.1–C.2, D.1–D.3, E.1–E.2, F). The cross-sectional area of the profile is 1468.8 mm^2^, while the unit weight of a 1 m long sample is 3.96 kg. The weight of the final product with a length of 6 m is 23.796 kg.

The development of the improved geometry of the new extrudate based on the concept of maximum weight reduction while maintaining the functionality of the elements, enabling their use in standard structures or as substitutes. As research has shown [16], thinning of the wall thickness reduces the strength and stiffness properties of the structure. For this reason, the reduction in strength and stiffness of the profiles due to the reduction in wall thicknesses was compensated by the use of the AA6005 alloy, which ensured superior mechanical properties. Unfortunately, this caused numerous difficulties in the extrusion process, including an increase in the extrusion force and a reduced flow rate causing reductions in extrusion velocity or surface quality, which was analyzed in this work and was an additional difficulty.

The profile was extruded from AA6063 alloy with the chemical composition given in Table 1.

Boundary conditions for numerical simulations and applied load values were defined based on the requirements of EN 1991, EN 1999 (Structural Eurocode) [17]. In this case, the snow load value was determined at the level of 0.9 kN/m^2^, which corresponds to a force of 900N directed vertically, perpendicularly to the exposed surface. A wind force load was applied at an angle of 45° with a value of 400 Pa per unit area, and the speed was specified as 30 m/min, as defined by the referenced standard.

To precisely and comprehensively test load conditions for various extrudate installation options in façade systems, two cases of fixed profile support were considered. 

In the first variant, the profile with a length of 3.5 m was fixed at the ends analogously to the classic beam bending test, where the load was applied in ½ of the length of the element.

In the second variant, a single profile with a length of 3.5 m was fixed with system brackets ensuring installation on the façade at a specific angle. In this case, the load was also applied at ½ of the length of the element perpendicularly to the external exposed surface of the profile.

The analyzed parameter was the maximum value of the profile deflection and the maximum value of the local mean stress occurring in the system. Table 3 presents the values of the determined parameter for the standard AA6063 alloy profile and the newly developed AA6005A alloy extrudate. The calculations used material models defined in the database for AA6063 and AA6005. The material parameters of the models are presented in Table 2. Material data originated from the Ansys Granta software database (2022 R1, Ansys Inc., Canonsburg, PA, USA). 

During numerical analyses, the value of the maximum deformation and the stress value were analyzed.

The boundary conditions defined for the simulation purposes correspond to the use parameters of finished products, determined on the basis of the standard EN 1991-1-3:2005 [17]. Among them, the following factors should be highlighted: snow load on the surface of 0.9 kN/m^2^ (a force directed vertically, perpendicular to the surface), wind speed of 30 m/s, inclination angle of 45°, value of force per unit area of 400 Pa, critical stress–strain state equal to 70% (maximum stress value in the profile below 0.7 yield point), and a profile length equal to 3500 mm.

### 2.2. Development of Die Design for a New Product

As part of our comprehensive research, technology for extrusion of the newly developed profile geometry from the AA6005A was developed. Numerical FEM simulations in QForm Extrusion were carried out. The die design was developed based on numerical simulations of the extrusion process of the newly developed profile.

Preparatory work and numerical simulations were carried out using the QForm Extrusion 3D (v.10.1.7, Micas Simulations Ltd., Oxford, UK). A special software module allows us to import a CAD model of tools and generate a mesh of finite elements on their surface and in the model volume. The module for performing numerical simulations consists of a pre-processor and a post-processor, providing visualization of the results [19,20]. The assumptions for numerical simulations are presented below.

The material model of the aluminum alloys was developed in plastometric tests of high-temperature compression on a Hegewald-Peschke Inspekt 100 (Hegewald & Peschke Meß- und Prüftechnik GmbH, Am Gründchen, Germany) testing machine with maximum force at level 100 kN. The machine has a dedicated heating chamber with a maximum temperature of 1200 °C in which samples are deformed.

The tests were carried out on samples of commercial billet material for the extrusion process in the form of cylinders with a diameter of Ø10 mm and a height of 15 mm with grease rolled into the middle on the front surfaces. The test was carried out for a wide range of sample deformation temperatures: 450 °C, 480 °C, 510 °C, 540 °C to 570 °C, and for constant strain values of 0.01, 0.1 and 1 s^−1^. The stress–strain curves obtained in the test for various temperature variants and strain rates were discretized, where factors were weighted and curves were fit to minimum standard deviation values RSQ, R2. Then, an approximation to the constitutive equation and determination of coefficients was carried out.

The concept of describing the flow stress using one curve makes it possible to empirically determine the rheological properties of the material in laboratory tests, e.g., in a high-temperature compression test. The influence of temperature during plastic deformation on the decrease in the value of flow stress should be taken into account. For this reason, determining the characteristics of the selected material at a wide range of temperatures and strain rates is crucial for the description of the rheological properties used in material extrusion tests [21,22,23].

The constitutive equation was the curve of flow stress as a function of actual strain, strain rate, and temperature described using the constitutive equation that describes the behavior of the material during plastic deformation regardless of the stress-state mechanism. The Hensel–Spittel constitutive equation was used to describe the rheological properties (1) [24].

(1) $$\sigma =A\cdot e^{m_{1}T}\cdot T^{m_{9}}\cdot \varepsilon^{m_{2}}\cdot e^{\frac{m_{4}}{\varepsilon }}\cdot {\left(1+\varepsilon \right)}^{m_{5}T}\cdot e^{m_{7}\varepsilon }\cdot {\dot{\varepsilon }}^{m_{3}}\cdot {\dot{\varepsilon }}^{m_{8}T}$$

where σ—yielding stress; T—temperature [°C]; ε—strain; $\dot{\varepsilon }$—strain rate [s^−1^]; A, m_1_-m_9_—coefficients of the equation. 

Discretized material in the form of the Hensel–Spittel constitutive equation was implemented in the database of the FEM software. This made it possible to perform numerical simulations of the AA6063 and AA6005A extrusion process using dedicated die models. In the case of standard technology, simulations were carried out using the standard die model for conventional manufacturing processes, on the basis of which the imperfections of the technology were determined. Based on the analysis of the simulation results of standard technology, an innovative die model was developed for extruding a thin-walled profile with reduced weight from the AA6005A alloy [25,26]. 

The result of the work is a porthole die designed for extruding a new profile and dedicated process parameters, including the heating temperature of the billet, die, and tool set, as well as the ram velocity.

### 2.3. Trial Tests and Quality Control

In the final stage of the research, the new technology was tested in industrial conditions with respect to the standard extrusion technology of conventional extrudates from AA6063. A set of parameters determined in FEM simulations was set; these parameters included ram velocity and billet heating temperature. This assumption allowed us to determine the accuracy of the results of numerical simulations. 

Industrial tests were carried out in manufacturing conditions on a horizontal hydraulic press with a maximum load of 35 MN with a Ø256 mm (10 inch) container (manufactured in Italy—Extral Technology S.R.L., Cividino, Italy).

During the tests, all process parameters were registered, including changes in pressure in the hydraulic system of the press as a measure of the change in the extrusion force, and metal flow speed as a change in the puller movement.

Trial tests of the new die (B) were carried out in industrial conditions with the same assumptions as those applied in extrusion tests using a standard die used in conventional technology (A). This made it possible to conduct a comparative analysis determining the impact of the die design on the tested process parameters. The same conditions for heating subsequent billets were used, but in the case of extrusion speed, this value was determined experimentally in tests in accordance with the adopted assumptions.

It was taken into account that the starting billet was extruded at a relatively low speed, which resulted in low temperature values of the beginning part of the extrudate. The ram velocity was therefore increased by controlling the pressure change curve in connection with the temperature of extrudates measured on the outlet of die. The pressure capabilities of the extrusion press and the low temperature of the extrudates allowed for an increase in the extrusion speed, simultaneously causing an increase in the temperature of the profile surface on the layout and ensuring uniform heating of the entire volume of the die.

Trial tests of the standard die (A) and the innovative die (B) were carried out using a measurement data acquisition system. Based on the data obtained, comparative charts were developed and were deemed necessary to analyze the impact of the die design on the process parameters. 

The pressure in the press system as a function of the ram displacement was measured by pressure sensors included in the press equipment. This parameter was monitored during the process and could be controlled on the press control panel. In turn, the speed and temperature of the press were measured on the press layout using external devices. The change in the extrudates velocity was defined by the displacement of the pulling system when the initial fragment of the press was placed in the puller clamp—a device designed to guide the extrudates evenly.

The temperature of the product was measured on the flat surface of the wall using a point laser pyrometer placed at the layout plate of the press.

The profiles extruded during industrial tests were the subject of comprehensive validation tests, including dimensional accuracy, microstructure analysis, and mechanical properties testing. 

Three-dimensional scanning was used to analyze the geometry of extrudates. This is a technology that records the shape of a physical object or environment to create a digital, three-dimensional model. The 3D scanning process involves measuring the distances and angles between the scanner and the object’s surface at multiple points, enabling the creation of a digital representation that accurately reflects the object’s size, shape, and texture [27]. The Atos Core 200 3D scanner (Carl Zeiss GOM Metrology GmbH, Braunschweig, Germany) was used for the tests. Gom Scan 2018 software (Carl Zeiss GOM Metrology GmbH, Braunschweig, Germany) was used to conduct and control the scanning process, and Gom Inspect 2018 software (Carl Zeiss GOM Metrology GmbH, Braunschweig, Germany) was used to analyze the 3D scanning results. The scope of the research included 3D scanning of extruded profiles in the final implementation of the standard profile (AA6063) and the new profile (AA6005A). After the 3D scanning process, surface models were generated from the point cloud. The 3D scans were then superimposed on the previously created CAD nominal models. For each analyzed sample, the values of the scan surface deviations from the nominal surface were generated in the form of deviations color maps and the value of average deviations of scans from nominal values (arithmetic mean and standard deviation). Additionally, for each sample, measurements were made for a specific group of dimensions (marked in Figure 1). The results were compared with the standard [28]. It was determined whether the tested measurement values were within the tolerances specified by the standard. Additionally, a correlation analysis of the results obtained after 3D scanning with the parameters of the extrusion process was performed. Origin Pro 2022 software (Originlab Corporation, Northampton, United States of America) was used for this purpose.

As part of the mechanical properties examination, a static tensile test was performed using a Hegewald & Peschke INSPEKT 100 kN testing machine (Hegewald & Peschke Meß- und Prüftechnik GmbH, Nossen, Germany). Tensile tests were carried out in accordance with the requirements of the standard [29]. Samples with a rectangular cross-section were used for tensile tests. The samples were stretched at a traverse movement speed of V = 5 mm/min at ambient temperature. Before the samples were placed in the jaws of the testing machine, the sample thickness was measured each time to determine the most accurate stress.

In the final stage of the research, the new technology was implemented in manufacturing conditions with defined velocity and temperature conditions, confirming the high accuracy of the results of numerical calculations.

## 3. Results

### 3.1. Development of Extrudate Cross-Section Geometry

The geometry of the new profile, which was improved in terms of utility and functionality, was developed based on numerical simulations of the static load on the profiles—both in testing the deflection of a single element and the entire system in conditions reflecting the real use construction.

The first stage of the research was the analysis of the stiffness of the AA6063 alloy profile and the identification of potential areas of optimization, taking into account the limitations of the extrusion process. The optimization assumed the maximum possible reduction in the unit weight of the profile while maintaining functionality, compatibility with the entire system, and the stiffness specified in the standard. As research has shown, the use of the more durable AA6005 alloy with a higher content of alloy components (especially Si and Mg) and mechanical properties provided compensation for the strength resulting from the reduction of the cross-sectional area.

**Figure 2:**
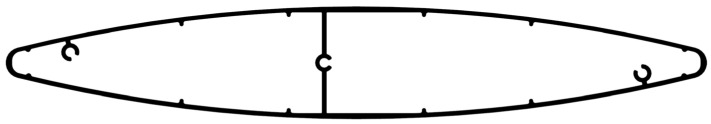
Cross-section geometry after the optimization procedure of the extrudate intended for the façade system.

Figure 2 shows a final cross-section of the extrudate developed as part of the optimization procedure. The unit weight of the profile was reduced by 0.526 kg, i.e., by 16% compared to the profile before optimization; this was affected by the weight of the 6 m long profile, which was only 20.640 kg. The profile wall thickness ranged from 1.6 mm to 1.8 mm.

**Figure 3:**
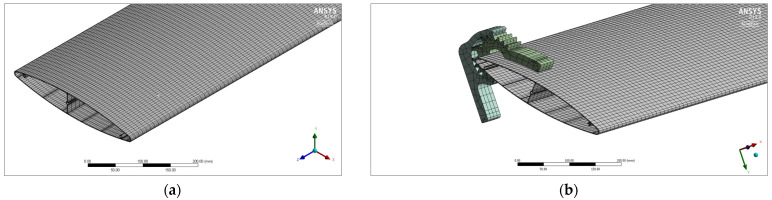
Finite element mesh on the surface of the developed profile in various test variants; (**a**) single profile; (**b**) profile in a system structure.

A finite element mesh (Figure 3) was prepared for both the standard profile and the developed profile. For the standard profile (AA6063), the number of mesh elements was 131.392 and the number of nodes was 796.542. Due to the greater degree of complexity of the mesh for the new profile (AA6005A), the number of mesh elements was 150.150 and the number of nodes was 836.834.

**Figure 4:**
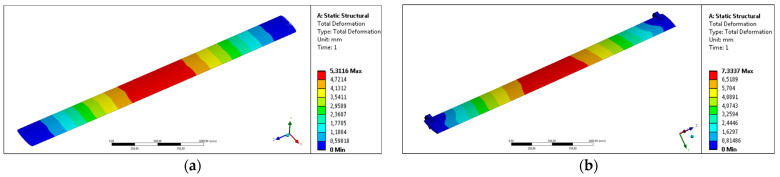
Results of static strength simulations of extrudates under various conditions; color-coded areas of total deformation [mm], (**a**) variant of calculation no. 1, (**b**) variant of calculation no. 2.

Figure 4 shows the results of numerical simulations for the maximum value of profile deflection and the maximum average value of the stress occurring in the system. The analyzed results of numerical simulations are qualitative distributions of selected parameters (maximum deflection, average stress) presented in the form of color-coded areas on the surfaces of the computational domain. 

**Figure 5:**
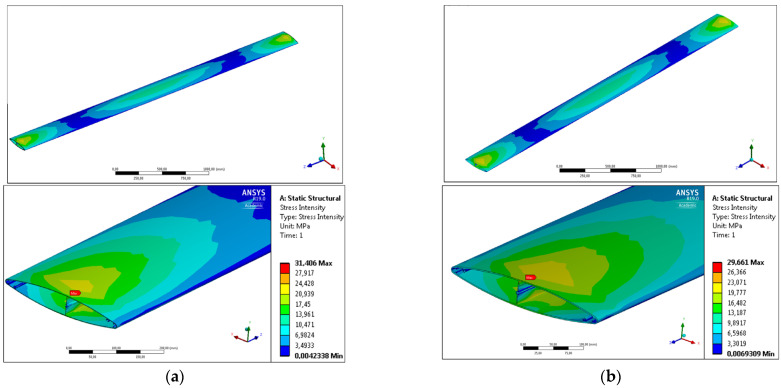
Calculation variant no. 1; (**a**) standard AA6063 extrudate, (**b**) new AA6005 extrudate.

Figure 5 shows the total deformation parameter distribution using color-coded areas on the surface of selected extrudates. The tendency of deformation of the system under specified load is similar both for calculation variants No. 1 (Figure 5a) and No. 2 (Figure 5b) and for various extrudates—the area of the largest deflection with a maximum value is observed in ½ of the profile length over a section of 500 mm. In the case of calculation variant No. 1 (where the ends of the profile are fixed directly through the cross-section surface), the distribution of the appropriate deformation zones is regular, especially the zone located next to the restraint area. Most importantly, the length of the maximum deformation section is the same. Only the determined local maximum values differ slightly—in the case of the developed profile with reduced weight made of the AA6005A alloy, this value is slightly higher (by only 0.002 mm). It can therefore be assumed that the impact of reducing the unit mass of the profile was effectively eliminated by the appropriate design—especially the stiffening protrusions located on the inner surface of the longest walls of the extrudate. 

In the case of calculation variant No. 2, where the profiles are fixed in the assembly system using rigid technological brackets, characteristic deformation zones are also observed along the product. In this case, the distribution of color-coded indicated value of parameters does not take a regular shape, but an increase in deflection is observed in the plane located on the unsupported zone of the handles (in 1/3 of the width of the profile cross-section—especially in the area of the vertical cross-section of the profile). Outside the support zone, an increase in deformation is observed, which is represented by an increase in the area of higher profile deflection values in characteristic areas. In each case, the area of maximum deformation is located at ½ of the length of the system, and the areas of minimum deformation are located at the system handles. In the case of the standard profile made of AA6063 alloy, the value of the total deformation is 0.477 higher than that of the structure using the new profile, which indicates a higher stiffness of the façade system constructed by new geometry extrudate with reduced weight and increased stiffness. 

The deformation of the profiles, which is the most important parameter from a practical point of view, whether in the restraint variant no. 1 or 2, is a direct effect of the stress distribution occurring in the system. For this reason, the influence of geometric changes and the alloy used on the distribution and value of stress intensity was also analyzed, both in the variant in which the profile was simply fixed at the ends of the cross-sections surface (variant No. 1) and in the case in which system brackets were used (variant No. 2).

**Figure 6:**
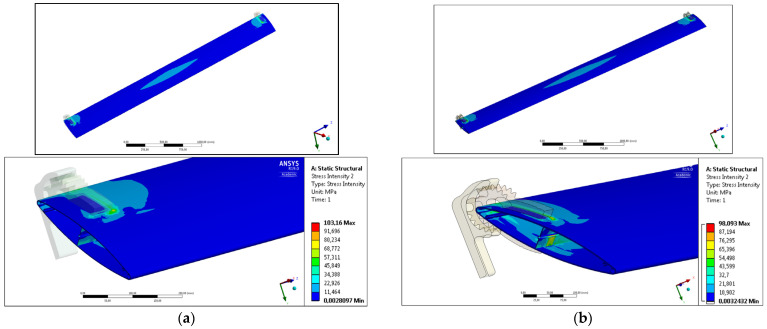
Calculation variant no. 2—profiles fixed in technological holders; (**a**) standard AA6063 extrudate, (**b**) new AA6005 extrudate.

In the first case of calculations for free restraint of profiles (Figure 5) characteristic areas of increased stress can be observed, especially in the central area located in ½ of the profile length and in the areas at the ends of the fixed profiles. There are no increased stresses in the areas between these parts. In the case of the central area, the occurring stress is directly responsible for the deflection of the profile in the direction consistent with the acting force, determining the maximum values of deflection. It should be emphasized, however, that the value of the maximum stress intensity was not observed in the central area, but in the areas located in the immediate vicinity of the ends of the profiles, where the restraint due to the complexity of the geometry and the cross-sectional area is the highest (Figure 6b). In the case of the standard profile, this value is 31.406 [MPa], while in the case of the new profile, this value is lower and amounts to 29.611, which demonstrates the higher stiffness and slenderness of the tested system.

In the case of test variant No. 2 (Figure 6b), the impact of product optimization on the stiffness of the system using system grips was determined.

In this case, two characteristic zones of deformation intensity are also observed: in the center, where the restraint is the smallest, which translates into the value of the deflection arrow; and in the restraint area, where, similarly to the calculation variant, the maximum value is observed. A lower value of maximum stress is observed by 5 MPa for the newly developed product with reduced weight made from the AA6005 alloy at the level of 98.093 MPa.

Based on the results obtained on the optimization path, a new geometry of the profile extruded from the AA6005 alloy with reduced wall thickness was developed. Special reinforcing elements located on the inner walls of the profile were used. As a consequence, the cross-sectional area was reduced by 16%, which translated into a reduction in the unit weight by 0.56 kg. The external geometry, as well as screw-ports and structural elements, have remained unchanged, ensuring compatibility with the entire standard façade systems. The summary of the results of numerical simulations of the structure load is presented in Table 3. 

As shown by the test results presented in Table 3, the new profile provides almost the same deflection at the level of 5.31 mm when testing the deflection of a single 3 m long extrudate fixed at the ends (variant No. 1). When using a new extrudate with a special geometry that ensures increased stiffness in the system development, a reduction in the maximum deflection value by 6% is observed. This effect is directly related to the distribution and the maximum value of stress intensity, which in both load variants is lower for the developed AA6005 extrudate with reduced unit mass. In the case of the calculation variant No. 1 of a single profile fixed at the ends, this value is lower by 6.1%; however, in the case of testing using system fixtures No. 2, this value is lower by only 1.7%.

### 3.2. Development of Extrusion Technology for a New Product

As a part of the project, a comprehensive technology of extrusion of a newly developed profile was developed, taking into account, among others, the most important consideration: the die design and the complete data of process parameters. For this purpose, numerical simulations of the technology used to produce standard profiles from the AA6063 alloy were performed. FEM simulations of the extrusion process were performed using a conventional bridge-chamber die (porthole die) with defined speed and temperature parameters. Based on the analysis of the results obtained and the defined inconveniences, a new, innovative die for extruding a newly developed profile with reduced wall thickness from the more demanding AA6005 alloy was designed. The above-mentioned modifications to the geometry of the extrudate and the use of a more durable alloy created additional difficulties. 

**Figure 7:**
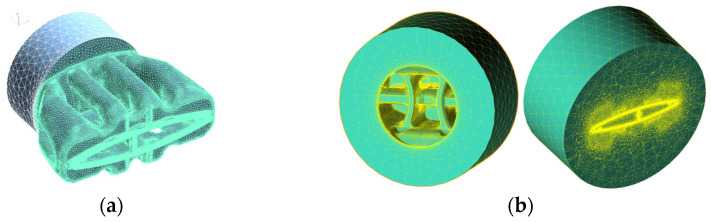
(**a**) FEM surface and volume mesh of calculation domain represent the internal volume of the die filled with aluminum, (**b**) FEM surface and volume mesh of calculation domain represent porthole die—front view (left), layout view (right).

Numerical simulations were performed using high-quality 3D models of both the computational domain of aluminum filling the die interior and the die set volume. In order to obtain qualitative results, a locally variable FEM mesh size was used. Appropriately, factors of the high-density mesh elements and element size factor were used for zones where plastic deformation intensifies. Especially in the area of creating metal flow to the prechamber, the coefficient of adaptation of profile geometry was used at the level of 0.45 and in the bearing zone at the level of 0.80 compared to the element size factor in the container at the coefficient level of 1.00 value (Figure 7).

**Figure 8:**
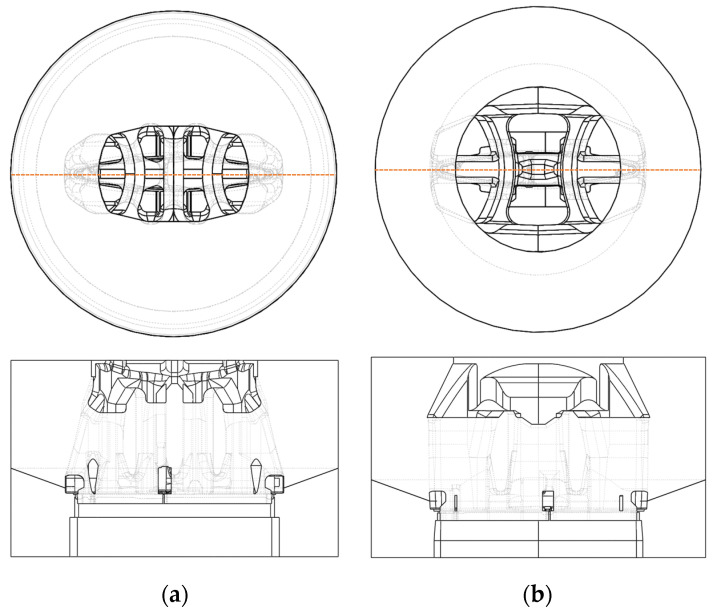
Three-dimensional porthole die with special spreader models; (**a**) for the standard profile (AA6063) and (**b**) for the new profile (AA6005A).

Figure 8a shows characteristic views of a conventional porthole die for extrusion AA6063, in view of the die inlet and in main cross-section. The entrance to the die is relatively small—with a small entrance diameter, three main bridges are used to hold the die core. Two external butterfly bridges ensure stiffness and increased metal flow to the outer areas of the bearing, which are potentially outside the billet entrance area (10” billet diameter). The use of a central vertical port theoretically increases the stiffness and strength of the structure, but on the other hand it significantly disturbs the flow of the metal, especially by intensifying the slower flow in the vertical crossbar. A structure in which a relatively short feeder is used is susceptible to deflection and deformation even in the initial phase of extrusion, when the metal fills the inlet channels.

**Figure 9:**
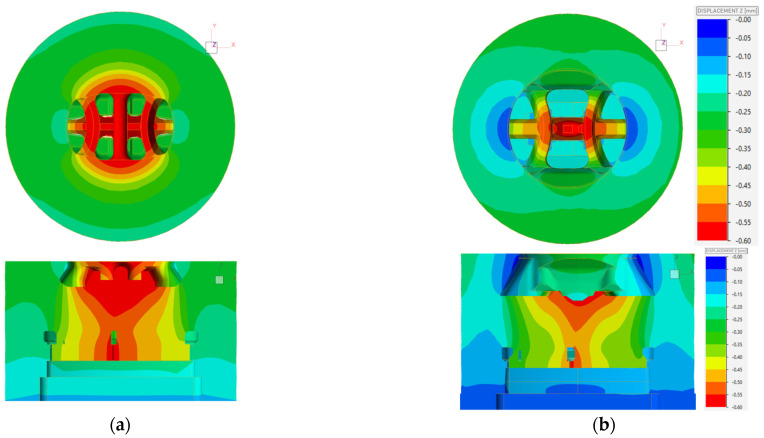
Color-coded areas of displacement of die volume in extrusion direction [Z] in view of die inlet (upper) and in cross section of die set; (**a**) standard die, (**b**) innovative die.

Quite a large deflection of the die in the central area was confirmed by the FEM simulation results presented in Figure 9a. The maximum deflection range of 0.9 mm is located almost throughout the entire cross-section of the die—especially in the central area of the die, in the entrance area, and in the calibrating area, which translates directly into the dimensional accuracy of the extruded profiles as well as the strength and durability of the die.

The new die uses a much larger opening of the inlet channels and a longer feeder, ensuring minimal resistance to metal flow into the die. Fewer “butterfly” bridges were used and the central bridge was eliminated. The increase in the strength of the structure was ensured by using larger bridge thicknesses. As a result, a much smaller area of maximum deflection was provided in relation to the conventional die determined in numerical simulations.

The fact that the die design directly affects the aluminum flow to the bearing and, at the same time, the die deflection velocity deviation in layout of die was taken into account during the analysis (Figure 9). The positive impact of the applied design limiting the elastic deflection of the die during the process in order to increase the uniformity of metal flow from the die opening and potential profile deformation was confirmed.

**Figure 10:**
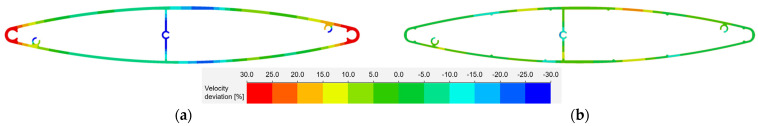
Color-coded percentage velocity deviation in die bearing cross section; (**a**) standard die, (**b**) innovative die.

Figure 10 shows percentage velocity deviation adjusted in cross section of bearing.

**Figure 11:**
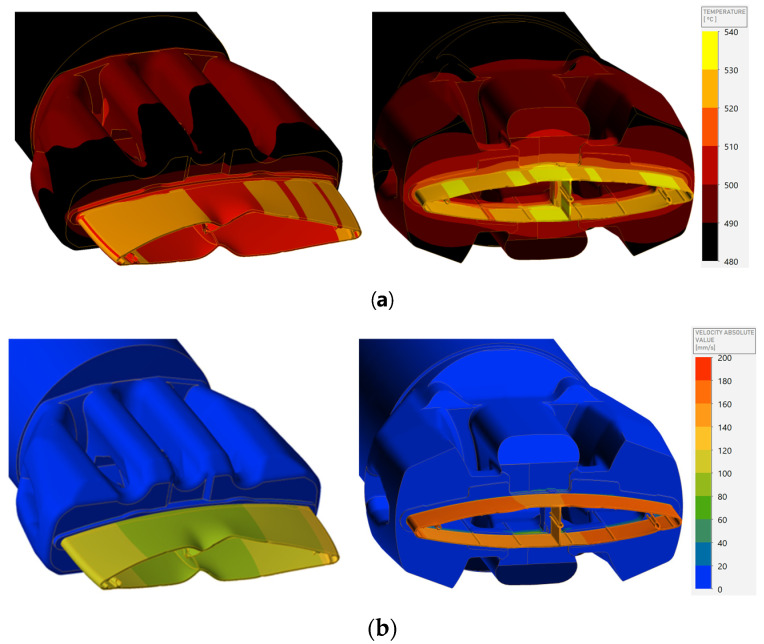
Numerical simulation results showing (**a**) flow and local velocity of metal and (**b**) temperature.

The way aluminum flows through the die translates directly into the value of the local outflow velocity and the flow kinetics, along with the potential deformation of the profile as well as the local temperature value. As shown in Figure 11, the outflow kinetics of the conventional profile are significantly disturbed due to the slower outflow in the vertical crossbar itself, which determines the deformation of the “nose piece” of the profile. This also causes differences in temperature values and in the area where the metal flows more slowly, a lower temperature of 510–520 °C is observed, while in the external areas this value is almost 10 °C higher. In the case of the new die (Figure 11b), a very uniform flow of metal is observed, even in the area of the crossbar and the outer corners of the profile, where achieving a successful flow is much more difficult. In the case of the innovative die where the bridges have been modified and flow in the center has been facilitated, an increase in temperature in the central area of the profile is observed due to the deliberate deceleration of the metal in this area. This value is almost 40 °C higher precisely because of this reason and due to the use of the 6005A alloy with higher values causing stress, which increases the temperature due to local friction.

**Figure 12:**
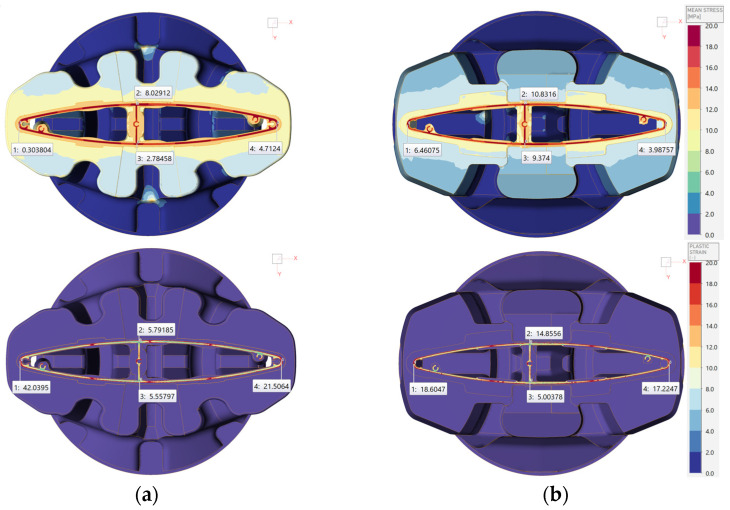
Numerical simulation results showing (**a**) mean stress (up—die A, down—die B) and (**b**) plastic strain.

The conditions determined in this way translate into the state of local stress and deformation in the bearing, which potentially translates into the quality of the extrudate. As shown in Figure 12, the value of the average stress in the characteristic areas and, at the same time, in the areas most exposed to cracking—such as the joints of vertical and horizontal walls—demonstrate the dominance of tensile stresses, as observed both in die A 6063 and in die B. 

As shown in Figure 12b, the new design, by uniformly providing a supply to the bearing, eliminated the local generation of plastic deformation occurring in die A.

### 3.3. Trial Tests

In the next stage of work, the simulation results were verified in implementation tests (trial tests) in production conditions. The main goal of this work was to test the developed technology by controlling process parameters and the quality of the produced extrudates. The tests also provided an opportunity to verify the results of numerical simulations, including the assumptions of the adopted boundary conditions and the developed material models.

**Figure 13:**
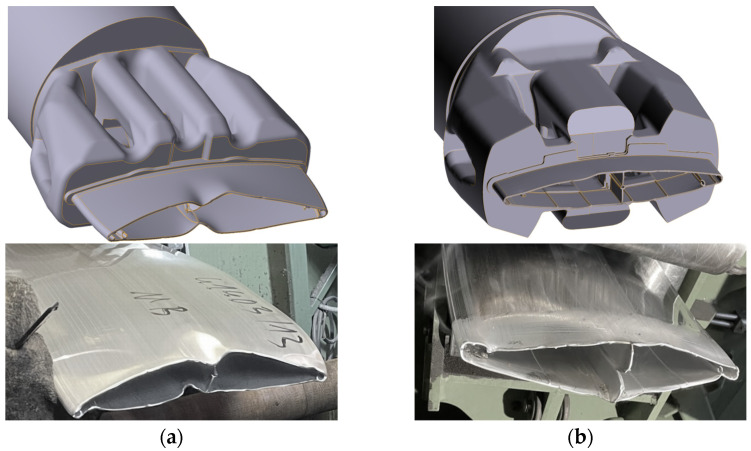
Comparison of simulation results (up) with trial tests (down)—geometry of the initial part of the extrudate indicating the uniformity of aluminum flow into the die bearing, (**a**) standard die (**b**) new innovative die.

The most intuitive and simple way to verify the accuracy of numerical simulations is to visually compare the initial outflow of a section of the profile from the die opening (commonly called the “nose piece”). The characteristic deformed initial part of the profile reflects the movement of aluminum flowing into the die, moving to the die clearance (bearing). The deformations are caused by the uneven intensity of local metal flow caused directly by the die structure. A properly designed die ensures an even flow of metal and is characterized by minimal geometric deviations in relation to the target shape of the profile. Figure 13 shows a comparison of the results of numerical simulations, illustrating the initial fragments of profiles compared with photographs of the beginnings of profiles taken during implementation tests of the first pilot ingot.

The analysis of the compiled photographs clearly indicates the high accuracy of numerical simulations. As can be seen in Figure 13a a standard profile extruded using conventional commercial technology from AA6063, there is a significant variation in the uniformity of metal flow from the die opening. Deformation of the walls in the area of the vertical crossbar is observed, indicating inadequate aluminum flow to the bearing in this critical place—the crossbar itself is deformed and insufficiently powered, which causes this area of the profile to flow much slower, causing ripples in the adjacent wall. At the same time, there is an excessively rapid flow of metal to the ends of the profiles, where there is a technological port with an increased cross-sectional area, which significantly facilitates the flow of metal. These described characteristic zones coincide both in the virtual model and in the presented Figure 13.

Also, in the case of the new profile with reduced wall thickness, which was made of AA6005A, the simulation accuracy is very high. It can be said that the uniform flow of the metal ensures uniform power supply to the bearing in almost the entire cross-section. Analogous zones disturbing the uniform flow of metal are observed, especially in the area of technological handles and the crossbar, where the cross-sectional surface is larger than that found locally in the thin-walled part of the profile—due to the small thickness of the walls, this is further intensified by the use of an alloy with a higher plasticizing stress. In the case of the virtual model, only a crack in ½ of the height of the vertical crossbar was not observed, which is due to the characteristics of the mathematical model and cannot be presented in a graphical interpretation. It should be stated that the results of numerical simulations correspond to the real behavior of metal in the extrusion process, which is visible in the comparative drawings presented, both on the virtual model and in the photographs from the implementation tests.

Implementation tests were carried out with the measurement data acquisition system. In this way, the force characteristics of the process were determined using the tested matrices.

**Figure 14:**
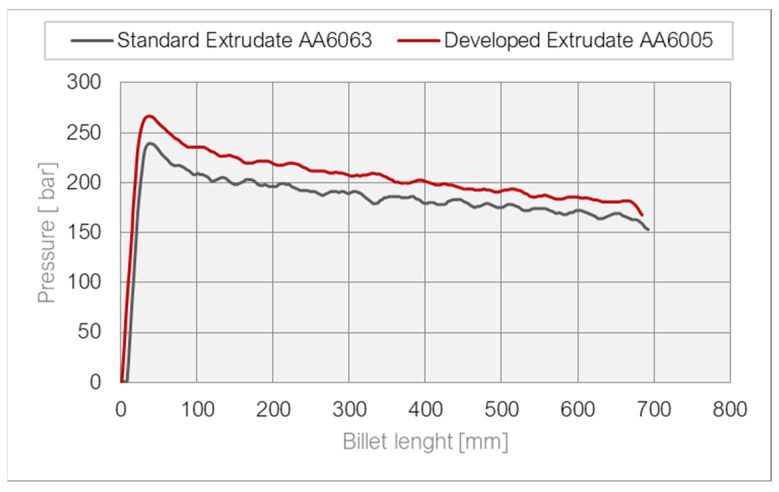
Force curve during bench press for AA6063 and AA6005.

The velocity of metal flow from the die opening was also examined in connection with the maximum temperature of the profile at the exit, which was a direct limit indicator (Figure 14). During the tests, the temperature value and the surface quality of the extrudates were controlled. The assumption was that the temperature limit of 560 °C should not be exceeded, which resulted in the occurrence of surface defects, scratches, and a heterogeneous structure of the exposed surface.

As shown in the graphs of the recorded data, in the case of tests of the newly designed die, the maximum force was almost 10% higher compared to the conventional die, which in this case is justified due to the extrusion of a harder alloy. The course of the curves is similar—a uniform increase to the maximum value is observed without a long acceleration period, which was a risk when using a long feeder and large-opening inlet channels.

**Figure 15:**
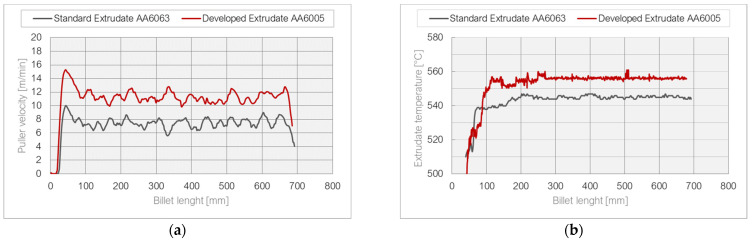
Graph of (**a**) puller speed for elements extruded from AA6063 and AA6005A, (**b**) temperature at the exit of the die for AA6063 and AA6005A.

It is important, however, that in the case of the new die, higher extrusion speeds were achieved by almost 2–5 m/min, which is an undoubted advantage, especially for a harder alloy. The comparative curves presented in the figure below clearly indicate that for the entire length of the billet, the speed was higher when the innovative die was used. This is also justified by the temperature values shown in Figure 15. Similarly, the temperature value is higher due to the use of the harder AA6005A and the higher extrusion speed. However, the value of 560 °C was not exceeded, above which surface defects may occur.

### 3.4. Product Validation

**Figure 16:**
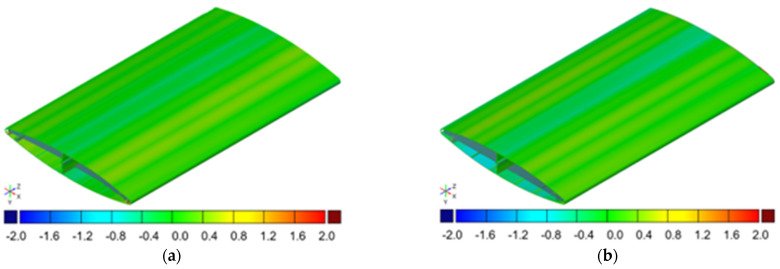
Three-dimensional scans of validation samples: (**a**) the standard profile (AA6063) and (**b**) the new profile (AA6005A).

Figure 16 shows sample 3D scanning results. Figure 16a shows a color map of the geometry deviations of the standard profile (AA6063), extruded with billet temperature at a level of 460 °C, at the maximum registered puller velocity at a level of 8 m/min. Figure 16b shows a color map of the geometry deviations of the new profile (AA6005A), extruded at the same billet temperature but with higher extrusion velocity at a level of 10 m/min. 

Based on the analysis of wall thickness measurements for a specific group of dimensions, compared with the tolerances presented in the standard [28], it was found that for all analyzed cases (12 samples) extruded as a standard profile AA6063, the analyzed dimensions meet the tolerances for 59% of cases. However, in the case of the new profile AA6005A (18 samples), the analyzed dimensions meet the tolerances in 99.5% of cases. 

Figure 16 shows the arithmetic means and the standard deviations of surface deviations of 3D scans from the nominal models. The results are presented for representative examples of the standard (AA6063) and new profiles (AA6005A).

**Figure 17:**
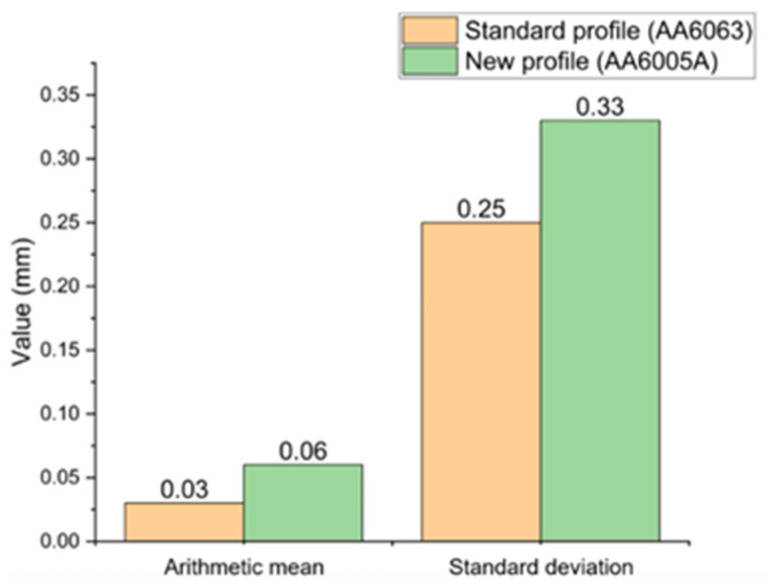
Arithmetic mean and standard deviation for 3D scans of representative profiles: standard (AA6063) and new (AA6005A).

Figure 17 shows example representative results of the arithmetic mean and standard deviation for the deviations in the scan surface of the standard profile (AA6063) and the new profile (AA6005A). The analysis was carried out for all tested cases of extruded profiles based on the standard profile—12 samples—as well as the new profile—18 samples. As can be observed in the case of representative samples (Figure 17), both the arithmetic mean and standard deviation are slightly higher for the new profile compared to the standard profile. For the remaining samples (throughout the entire research scope, not shown in Figure 17), the arithmetic mean of the deviation, both for the standard and new profiles, was in the range from 0 mm to −0.07 mm. The largest standard deviation for comparing the scan surfaces to nominal values in the entire research range was 0.54 mm. However, the smallest standard deviation was 0.05 mm. The standard deviation was smaller for the new profile, so the results indicate data grouped around the mean. In the case of the standard profile, the standard deviation indicated highly scattered results.

**Figure 18:**
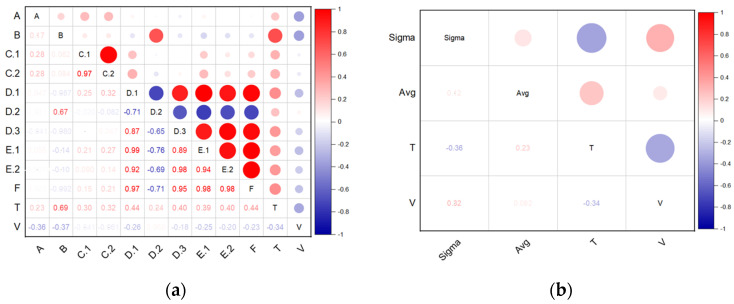
Results of correlation tests; (**a**) between the dimensions selected for testing and the parameters of the extrusion process. The letters A-F indicate the selected group of 10 measurements, while the letter T indicates the temperature and the letter V indicates the extrusion speed, (**b**) between arithmetic average, standard deviation average and process parameters. Sigma is the standard deviation; avg. is the arithmetic mean.

Figure 18a shows the results of testing the correlation between wall thickness measurements for a specific group of dimensions (shown in Figure 1), compared with the tolerances presented in the standard [28], and the process parameters (temperature and extrusion speed). Figure 18b shows the results of the correlation study between arithmetic means and standard deviation and process parameters (temperature and extrusion speed).

The higher the positive correlation index (i.e., the closer it is to one), the more intense the red color. The correlation is both positive and negative. In the first case, this means that as the value of one tested feature increases, the value of the other increases, while in the second case, it means that as one variable increases/decreases, the other variable behaves inversely and decreases or increases accordingly. The strongest positive correlation between the examined dimensions and the process parameters (Figure 18a) can be observed for dimension B and temperature (0.69). No strong negative correlations were observed. In the case of the analysis of the correlation between the arithmetic mean and standard deviation and the process parameters (Figure 18b), no significant correlations were observed between the tested means and the process parameters. In all cases, the correlation was below 0.4.

**Figure 19:**
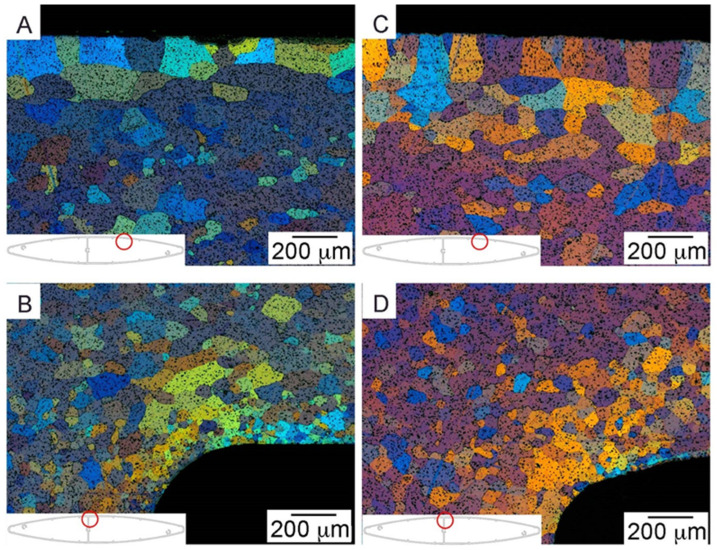
The microstructure of the profile: (**A**,**B**) made of alloy 6005A; (**C**,**D**) made of alloy 6063.

Figure 19a,b show the microstructure of a profile made of alloy 6005A, while Figure 19c,d show the microstructure of a profile obtained from alloy 6063. The photos were taken on cross-sections of extruded profiles in places marked with red pins.

Two types of phases can be distinguished in the 6xxx series alloys: those containing magnesium and silicon and those containing aluminum, iron, manganese, and silicon. Precipitates rich in Mg and Si are Mg_2_Si phases, the presence of which can also be confirmed in scientific research articles [29,30,31]. Phases rich in iron, manganese and silicon are most often Al(FeMn)Si phases [32,33]. Literature data indicate that in this type of alloys, depending on their chemical composition, there are phases with different stoichiometric compositions (Al(FeMn)Si, Al_9_Mn_3_Si, Al_5_FeSi). Microscopic examinations showed no discrepancies in the obtained profiles. In the case of the 6005A alloy profile, the average grain diameter was 90 μm and the peripheral coarse grain (PCG)was 125 μm. In the case of the 6063 alloy profile, the average grain diameter was 126 μm and the PCG was 205 μm. Peripheral coarse grain in aluminum alloys is caused by too high a heating temperature for the supersaturation process and too long spent at high temperature. The authors of [34] examined the influence of the ram speed on the thickness of the PCG in the AA 6063 alloy. The research showed that the thickness of the coarse-grained rim increased with the ram speed. The reason for the increase in the coarse-grained rim is because the deformation temperature increases with increasing speed, and the energy stored in the samples from the surface towards the center increases with increasing speed. The energy stored at the periphery, released as grain growth after extrusion, is called static recrystallization, and is followed by peripheral or abnormal grain growth [35]. Similar results were reported by the authors of a paper in which an electron backscatter diffraction (EBSD) technique was used to analyze the orientation of deformed microstructures for extrusions from aluminum alloys of the 6xxx series [3].

Table 4 shows the results of mechanical properties tests for the standard profile (AA6063) and the new profile (AA6005A) compared to data from the standard [36].

The results of mechanical properties tests for the 6063 and 6005A alloy profiles are presented in Table 4. The average tensile strength of the 6063 alloy profile was 240 MPa, while the elongation reached 13.5%. The tensile strength of the 6005A alloy profiles was 282 MPa, while the elongation reached 11.2%. These values meet the requirements set out in the standard [37].

In order to confirm the repeatability of the results, the test was carried out on five samples for each variant. The values given are average values where the maximum value of the standard deviation does not exceed value of 3.8 of the obtained results. The higher strength properties and hardness of the 6005A alloy may be caused by the content of alloy additives as well as the smaller average grain diameter compared to the 6063 series alloy. According to the Hall–Petch relation, the increase in strength properties is caused by the strengthening coming from the grain boundaries [38].

## 4. Summary

The utilization of AA 6005A alloy, which is characterized by higher strength compared to the commonly used AA 6063, has proved to be a key factor in the profile shape modification process. Due to the higher flow stress value, it is necessary to use advanced extrusion technology to produce high-quality, thin-walled, and low-weight extrudates with high production efficiency. Overcoming the technological and economic barrier and using AA6005 for thin-walled, wide extrudates created new possibilities for even more advanced changes in construction, allowing for the reduction of unit weight by wall thickness while maintaining the desired strength parameters.

Despite the challenges associated with the higher strength of aluminum alloys, a properly designed die construction has significantly contributed to the increased efficiency of the extrusion process. Optimization of this issue is crucial, especially in the production of elements with more complex shapes, where maintaining strength while simultaneously reducing wall thickness is crucial for achieving desired effects.

Precise adjustment of material parameters and faithful reconstruction of the extrusion process for various profile shapes are necessary to achieve high compliance with the results of conducted technological trials. This methodology is key to effectively optimizing geometry and the production process, which is of great importance for a wide range of industrially produced elements.

Additionally, the use of 3D scanning allows for precise assessment of profile geometry, and in the case of elements made of AA 6005A alloy, satisfactory geometric quality has been observed, with dimensional deviations falling within dimensional tolerance ranges. This confirms not only the precision of the process but also its consistency and repeatability.

Ultimately, maintaining the strength of the extruded profile despite reducing wall thickness allows for a significant reduction in the consumption of aluminum required for production. This is beneficial both from an economic perspective, through the reduction of raw material costs, and from an environmental perspective, through the reduction of material consumption. This balanced production strategy has tremendous potential in achieving both economic and environmental efficiency in the manufacturing industry.

Continuous research, analysis, and improvement of processes are crucial for maintaining a competitive advantage in today’s dynamic business environment, especially in industries that are constantly evolving and requiring innovation. Therefore, understanding and continuously improving extrusion processes are integral elements of industrial strategies aimed at achieving higher quality, greater efficiency, and sustainable development.

The experimental findings have indeed fulfilled the anticipated expectations, though achieving this outcome necessitated a considerable amount of effort in refining the correct geometry and extrusion process parameters. Precision in determining the ideal geometry and meticulously adjusting the extrusion parameters were pivotal in attaining the desired results. Despite the substantial challenges faced along the way, the commitment to accuracy and thoroughness ultimately paid off, demonstrating the importance of meticulous planning and perseverance in experimental research. The obtained results of the geometric analyses showed that altering the geometry of the component and the new aluminum alloy used did not have a negative impact on the quality of the produced items, and dimensional deviations did not exceed the specified geometric tolerances.

It should also be noted that the development of advanced technology supported by FEM static structural simulation and the extrusion process enables relatively quick and more efficient development of new products and manufacturing technologies without the need to produce material preliminary versions, which significantly reduces the cost of research and of introducing aluminum to the environment.

As part of our research project, research was carried out on the influence of the speed and intensity of extrudate cooling on the press layout on the microstructure and mechanical properties after the artificial aging process.

The subject of our research was various methods of cooling extrudates on the production line, such as cooling in a waterbox, with a water spray or in a nitrogen atmosphere. This created even greater opportunities to increase the mechanical properties of extrudates and, consequently, reduce the weight of extrudates intended for facade systems. The developed die design concept can also contribute to increasing the possibility of extrusion of thin-walled, wide profiles with economically justified efficiency.

In the future, there are plans to continue the research based on the methodology presented in the publication for extruded profiles with different shapes. Switching to a stronger alloy allows for the thickness of the walls to be reduced, with a consequent decrease in the weight of the component. However, with increased alloy hardness comes greater difficulty in achieving satisfactory results in terms of component quality, necessitating significant changes in process parameters. The amount of aluminum needed to manufacture the unit weight of the profile decreased by 16%, resulting in a corresponding 6% estimated decrease in material costs due to differences in the price of the aluminum alloys used. Importantly, this translates into a total reduction in carbon emissions, and additionally, together with the use of special low carbon aluminum billets produced from renewable energy and recycled aluminum, it will allow the carbon footprint to be reduced by up to 25% compared to the currently used technology.

## 5. Patents

The developed profile geometry is the subject of utility model application number P.447079 WIPO ST 10/C PL447079.

## Figures and Tables

**Table 1:** Chemical composition of materials intended for research (wt. %).

Alloy	Mg	Si	Mn	Fe	Cu	Zn	Ti	Ga	V	Al
6063	0.5	0.45	0.03	0.21	0.02	0.15	0.13	0.1	0.1	balance
6005A	0.57	0.71	0.15	0.16	0.03	0.1	0.1	0.1	0.1	balance

**Table 2:** Material parameters of the models adopted for numerical analyses [18].

Alloy	6063 T6	6005A T6	Unit
Density	2700	2700	kg·mm^−3^
Young’s modulus	68 900	69 000	MPa
Poisson’s ratio	0.33	0.33	-
Bulk modulus	56917	67647	MPa
Shear modulus	26269	25940	MPa
YTS	214	250	MPa
UTS	241	300	MPa

**Table 3:** Summary of numerical simulation results for the standard profile (AA6063) and the new profile (AA6005A).

**Maximum Deflection Value (mm)**		
Variant of calculation	Standard profile AA6063	New profile AA6005A
No. 1—Single extrudate	5.31	5.31
No. 2—System installation	7.78	7.33
**Stress intensity (MPa)**		
Variant of calculation	Standard profile AA6063	New profile AA6005A
No. 1	31.4	29.6
No. 2	103.16	98.09

**Table 4:** Results of testing the mechanical properties of AA6063 and AA6005A alloys.

Profile	Test Results		Standard Data	
	R_m_ (MPa)	A (%)	R_m_ (MPa)	A (%)
AA6063	240	13.5	215	8
AA6005A	282	11.2	255	8

## Data Availability

Data are contained within the article.
